# Multi-Objective Robust Design Optimization for Crashworthiness Enhancement of Hybrid 2D Triaxially Braided Composite Tube Using Evolutionary Algorithms

**DOI:** 10.3390/polym16172457

**Published:** 2024-08-29

**Authors:** Dongyang Sun, Yudu Jiao, Yuanhao Tian, Youkun Gong, Leilei Li, Huiming Ning

**Affiliations:** 1 School of Naval Architecture and Ocean Engineering, Jiangsu University of Science and Technology, Zhenjiang 212003, China; sundongyang@cqu.edu.cn; 2 Laboratory of Science and Technology on Marine Navigation and Control, China State Shipbuilding Corporation, Tianjin 300131, China; lill@cqu.edu.cn; 3 College of Aerospace Engineering, Chongqing University, Chongqing 400044, China; 201831131022@cqu.edu.cn; 4 Southwest Technology and Engineering Research Institute, Chongqing 400039, China; yuanhaotian@cqu.edu.cn; 5 Chongqing Polycomp International Corporation, Chongqing 400084, China; gongkun520@126.com

**Keywords:** two-dimensional tri-axial braided composites, crashworthiness, surrogate methods, genetic algorithm, multi-objective robust optimization

## Abstract

An innovative optimal design framework is developed aiming at enhancing the crashworthiness while ensuring the lightweight design of a hybrid two-dimensional triaxial braided composite (2DTBC) tube, drawing insights from the mesostructure of the composite material. To achieve these goals, we first compile the essential mechanical properties of the 2DTBC using a concentric cylinder model (CCM) and an analytical laminate model. Subsequently, a kriging surrogate model to elucidate the intricate relationship between design variables and macroscopic crashworthiness is developed and validated. Finally, employing multi-objective evolutionary optimization, we identify Pareto optimal solutions, highlighting that reducing the total fiber volume and increasing the glass fiber content in the total fiber volume are crucial for optimal crashworthiness and the lightweight design of the hybrid 2DTBC tube. By integrating advanced predictive modeling techniques with multi-objective evolutionary optimization, the proposed approach not only sheds light on the fundamental principles governing the crashworthiness of hybrid 2DTBC but also provides valuable insights for the design of robust and lightweight composite structures.

## 1. Introduction

To tackle concerns regarding sustainable development and transportation safety, reducing fuel consumption and enhancing occupant safety are crucial in industries, such as aerospace and automotive. One of the most effective methods to achieve these goals is through the reduction of structural weight [1]. Fiber reinforced polymers (FRPs) are commonly utilized for this purpose due to their high specific strength, stiffness, and excellent energy absorption during crash events [2]. Consequently, the rational and optimal design of composite structures has emerged as a popular area of research to enhance performance and decrease weight [3,4,5,6].

Numerous studies have explored the optimal design of laminated composite structures, with scholars focusing on optimizing structural parameters, like the layup angle, sequence, thickness, and material type, to improve performance aspects, such as impact resistance and energy absorption. For instance, Kim et al. [7] utilized a micro-genetic algorithm (μGA) to design a hybrid glass–carbon composite beam, resulting in a 33% weight reduction and improved crash performance. Aiming at maximizing the specific energy absorption (SEA) and minimizing the peak impact load of tape sinusoidal composite structures under axial impact loading, Duan et al. [8] optimized the geometry of specimens through single and multi-objective optimizations to enhance SEA and minimize the peak impact load (PIL) for band sinusoidal composite structures under axial impact loading. To reduce the weight and head injury criterion (HIC) while increasing energy absorption, Zeng et al. [9] optimized the thickness, material constituent, and layup configuration of composite bumper beams using the fruit fly optimization algorithm (FOA). Paz et al. [10] designed a hybrid square hollow steel tube filled with a honeycomb structure made of glass-fiber reinforced polyamide using a surrogate-based optimization technique to reduce the weight, increase the absorbed energy, and decrease the peak impact load.

Braided composites are becoming increasing popular as they allow for good structural integrity, damage tolerance, and design flexibility compared to laminated composite [11,12]. Two-dimensional triaxial braided composites (2DTBCs) are a specific type of braided composites that have wide application prospects in aerospace and automotive applications, such as the fan sealing system of the GEnx engine. In recent years, hybrid 2DTBC composites, which combine two different fiber-reinforcement phases, namely carbon and glass fibers in a matrix, have been developed. By blending a specific proportion of glass fibers with carbon fibers, these composites reduce material costs while simultaneously enhancing impact toughness, attributed to the superior toughness of glass fibers. Accurate prediction of the mechanical properties of 2DTBCs is essential for the further analysis and design of structural components. Quek et al. [13] evaluated the stiffness matrix of a 2DTBC based on the concentric cylinder model (CCM) [14], considering the curling effect of the biased fiber bundles. Ye et al. [15] employed CCM and the rule of mixture (ROM) [16] to determine the overall stiffness of a hybrid 2DTBC. For the strength prediction of 2DTBCs, Xiao et al. [17] proposed an approximate method for predicting the strength of a 2DTBC that incorporates the in-plane periodic mesostructure of the textile composite in finite element models. Bai et al. [18] developed a micromechanical model to analyze the in-plane shear and tensile strengths of 2DTBCs and explored the interaction effect among angularly interlaced yarns.

Although there have been numerous research studies on the optimal design of unidirectional fiber reinforced composites, there are few studies on optimizing structures produced by a 2DTBC from the perspective of material microstructures. Yang et al. [19] optimized a 2DTBC hollow pillar by minimizing the weight and cost under the constraint of compressive load and buckling load based on probability theory. However, only elastic analysis is performed during optimization, and the failure model of the 2DTBC is not considered. In this paper, an analytical laminate model of a 2DTBC has been adopted to predict both the elastic and strength properties of the hybrid 2DTBC, in which the axial reinforcement is carbon tow and braided reinforcement is composed of glass tow. And then we proposed a framework that can investigate the macroscopic crashworthiness capacity of a composite tube based on the mesostructure of the hybrid 2DTBC. Using the total fiber volume fraction, the relative volume fraction of the carbon fiber in the total fiber volume fraction, and the braid angle as the design variables, two typical optimization problems are described, including the crashworthiness issues and lightweight design. Finally, the two optimization problems were solved with Pareto optimal solutions using multi-objective evolutionary algorithms.

## 2. Analytical Model of 2DTBC

### 2.1. Local Stiffness Analysis

**Figure 1:**
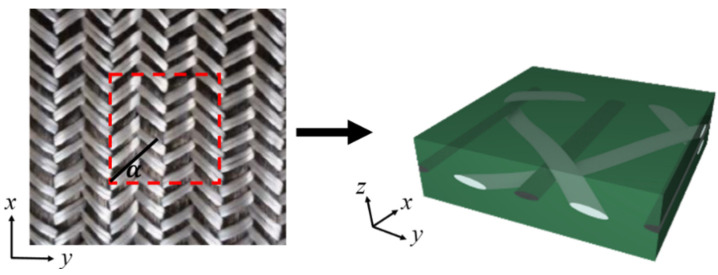
Representative unit cell of 2DTBC.

**Figure 2:**
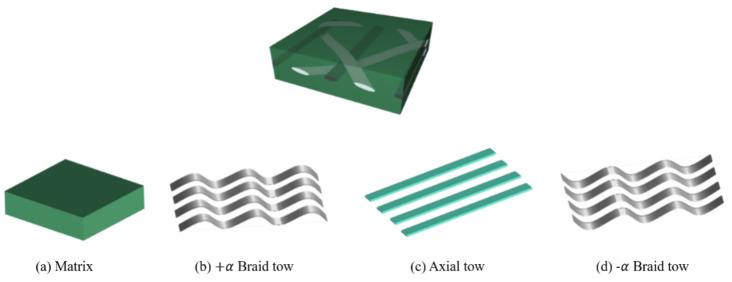
Four constituents of 2DTBC.

Since the braided composite has periodic boundary conditions, the smallest representative unit cell (RUC) is selected as the research object. The analysis of the 2DTBC is based on RUC as shown in Figure 1. The axis-x represents the direction of the axial tow, the axis-y denotes the transverse direction, and axis-z is perpendicular to the plane of the 2DTBC. In this work, the hybrid 2DTBC is considered to be composed of four parts depicted in Figure 2. Axial reinforcement is carbon tow (Figure 2c), braided reinforcement is constructed with two sets of glass tows (Figure 2b,d), and the remaining one represents the matrix (Figure 2a).

The starting point for the analysis is the concentric cylinder model (CCM) for a transversely isotropic composite. The CCM model provides five independent elastic constants: longitudinal Young’s modulus $E_{11}$, transverse bulk modulus $k_{23}$, longitudinal shear modulus $G_{12}$, longitudinal Poisson’s ratio $v_{12}$, and transverse shear modulus $G_{23}$. The first four are given as follows based on the two-phase CCM [20,21]:

(1) $$k_{23}=k_{m}+\frac{V_{f}}{1/\left(k_{23f}-k_{m}\right)+V_{m}/\left(k_{m}+G_{m}\right)}$$

(2) $$E_{11}=E_{11f}V_{f}+E_{m}V_{m}+\frac{4V_{f}V_{m}{\left(v_{12f}-v_{m}\right)}^{2}}{V_{f}/k_{m}+V_{m}/k_{23f}+1/G_{m}}$$

(3) $$v_{12}=v_{12f}V_{f}+v_{m}V_{m}+\frac{V_{f}V_{m}\left(v_{12f}-v_{m}\right)\left(1/k_{m}-1/k_{23f}\right)}{V_{f}/k_{m}+V_{m}/k_{23f}+1/G_{m}}$$

(4) $$G_{12}=G_{m}\frac{G_{12f}\left(1+V_{f}\right)+G_{m}V_{m}}{G_{m}\left(1+V_{f}\right)+G_{12f}V_{m}}$$

where E is Young’s modulus, k is the transverse bulk modulus, v is the Poisson’s ratio, and G is the shear modulus. V is the volume fraction, $V_{f}+V_{m}=1$, subscripts *m* and *f* represent the matrix and fiber, respectively. The transverse shear modulus can be solved using a quadratic equation according to the three-phase CCM:

(5) $$A{\left(\frac{G_{23}}{G_{m}}\right)}^{2}+B\left(\frac{G_{23}}{G_{m}}\right)+D=0$$

where

(6) $$\begin{array}{c} D=3V_{f}V_{m}^{2}\left(\frac{G_{23f}}{G_{m}}-1\right)\left(\frac{G_{23f}}{G_{m}}+\zeta_{f}\right)+\left[\frac{G_{23f}}{G_{m}}\zeta_{m}+\left(\frac{G_{23f}}{G_{m}}-1\right)V_{f}+1\right]][\frac{G_{23f}}{G_{m}}\\ +\zeta_{f}+\left(\frac{G_{23f}}{G_{m}}\zeta_{m}-\zeta_{f}\right)V_{f}^{3}] \end{array}$$

(7) $$\begin{array}{l} B=-6V_{f}V_{m}^{2}(\frac{G_{23f}}{G_{m}}-1)(\frac{G_{23f}}{G_{m}}+\zeta_{f})\\ +[\frac{G_{23f}}{G_{m}}\zeta_{m}+(\frac{G_{23f}}{G_{m}}-1)V_{f}+1]\\ \times [(\zeta_{m}-1)(\frac{G_{23f}}{G_{m}}+\zeta_{f})-2V_{f}^{3}(\frac{G_{23f}}{G_{m}}\zeta_{m}-\zeta_{f})]\\ +V_{f}(\zeta_{m}+1)(\frac{G_{23f}}{G_{m}}-1)[\frac{G_{23f}}{G_{m}}+\zeta_{f}+V_{f}^{3}(\frac{G_{23f}}{G_{m}}\zeta_{m}-\zeta_{f})] \end{array}$$

(8) $$\begin{array}{l} A=3V_{f}V_{m}^{2}(\frac{G_{23f}}{G_{m}}-1)(\frac{G_{23f}}{G_{m}}+\zeta_{f})\\ +[\frac{G_{23f}}{G_{m}}\zeta_{m}+\zeta_{f}\zeta_{m}-(\frac{G_{23f}}{G_{m}}\zeta_{m}-\zeta_{f})V_{f}^{3}]\\ \times [\zeta_{m}V_{f}(\frac{G_{23f}}{G_{m}}-1)-(\frac{G_{23f}}{G_{m}}\zeta_{m}+1)] \end{array}$$

(9) $$\zeta_{m}=3-4\upsilon_{m}$$

(10) $$\zeta_{f}=3-4\upsilon_{23f}$$

However, $E_{22}$ is not directly provided in the CCM model. Quek et al. proposed a modified version of ROM [22,23] to calculate transverse Young’s modulus $E_{22}$:

(11) $$\frac{1}{E_{22}}=\frac{\eta_{f}V_{f}}{E_{22f}}+\frac{\eta_{m}V_{m}}{E_{m}}$$

where

(12) $$\eta_{f}=\frac{E_{11f}V_{f}+\left(E_{m}\left(1-v_{12f}v_{21f}\right)+E_{11f}v_{m}v_{21f}\right)V_{m}}{E_{11f}V_{f}+E_{m}V_{m}}$$

(13) $$\eta_{m}=\frac{E_{m}V_{m}+\left(E_{11f}\left(1-v_{m}^{2}\right)+E_{m}\left(1-v_{m}v_{12f}\right)\right)V_{f}}{E_{11f}V_{f}+E_{m}V_{m}}$$

In Quek’s model, the other four elastic constants are calculated as the same as the original concentric cylinder model using Equations (2)–(5). Now the local stiffness matrix of axial tow is obtained by combining CCM and Quek’s model.

### 2.2. Macroscopic Stiffness

**Figure 3:**
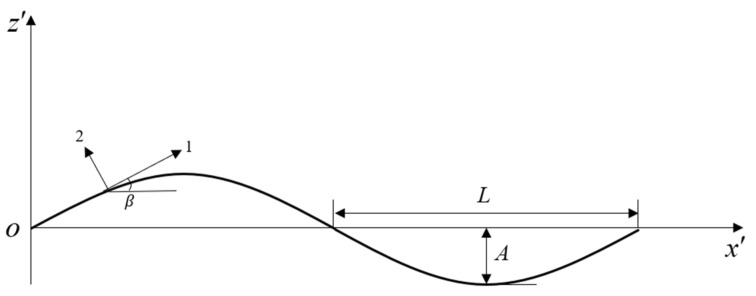
Undulating curve of braid tows.

In the braiding process, braid tows and axial tows interlace at an angle. The stiffness of the two braid tows can be determined by taking the crimp angle and braid angle effect into account and integration over one wavelength. To make the crimp angle clear, the braid tow can be considered a sinusoidal undulating curve with amplitude A and wavelength 2L (Figure 3).

(14) $$z=A\sin\left(\frac{\pi x}{L}\right)$$

The crimp angle is calculated as

(15) $$\tan(\beta )=\frac{\pi A}{L}\cos\left(\frac{\pi x}{L}\right)$$

The stiffness of the braid tow is given as the integral along the wavelength:

(16) $$[C]=\frac{1}{2L}\int 2L{\left[{\hat{T}}_{1}\right]}^{-1}\left[C_{0}\right]\left[{\hat{T}}_{2}\right]dx$$

The transformation matrices are given as

(17) $$\left[{\hat{T}}_{1}\right]=\left[\begin{array}{cccccc} {\hat{m}}^{2} & 0 & {\hat{n}}^{2} & 0 & 2\hat{m}\hat{n} & 0\\ 0 & 1 & 0 & 0 & 0 & 0\\ {\hat{n}}^{2} & 0 & {\hat{m}}^{2} & 0 & -2\hat{m}\hat{n} & 0\\ 0 & 0 & 0 & \hat{m} & 0 & -\hat{n}\\ -\hat{m}\hat{n} & 0 & \hat{m}\hat{n} & 0 & {\hat{m}}^{2}-{\hat{n}}^{2} & 0\\ 0 & 0 & 0 & \hat{n} & 0 & \hat{m} \end{array}\right]$$

(18) $$\left[{\hat{T}}_{2}\right]=\left[\begin{array}{cccccc} {\hat{m}}^{2} & 0 & {\hat{n}}^{2} & 0 & \hat{m}\hat{n} & 0\\ 0 & 1 & 0 & 0 & 0 & 0\\ {\hat{n}}^{2} & 0 & {\hat{m}}^{2} & 0 & -\hat{m}\hat{n} & 0\\ 0 & 0 & 0 & \hat{m} & 0 & -\hat{n}\\ -2\hat{m}\hat{n} & 0 & 2\hat{m}\hat{n} & 0 & {\hat{m}}^{2}-{\hat{n}}^{2} & 0\\ 0 & 0 & 0 & \hat{n} & 0 & \hat{m} \end{array}\right]$$

where

(19) $$\hat{m}=\cos(\beta ),\hat{n}=\sin(\beta )$$

The local averaged stiffness [C] in the system ($x^{\prime }$$y^{\prime }$$z^{\prime }$) is transformed into the global coordinate system (xyz), through braid angle α. The global stiffness matrix is given by

(20) $$\left[C_{\alpha }\right]={\left[T_{1}\right]}^{-1}[C]\left[T_{2}\right]$$

where the transformation matrices $T_{1}$ and $T_{2}$ are given by

(21) $$\left[T_{1}\right]=\left[\begin{array}{cccccc} m^{2} & n^{2} & 0 & 0 & 0 & 2mn\\ n^{2} & m^{2} & 0 & 0 & 0 & -2mn\\ 0 & 0 & 1 & 0 & 0 & 0\\ 0 & 0 & 0 & m & -n & 0\\ 0 & 0 & 0 & n & m & 0\\ -mn & mn & 0 & 0 & 0 & m^{2}-n^{2} \end{array}\right]$$

(22) $$\left[T_{2}\right]=\left[\begin{array}{cccccc} m^{2} & n^{2} & 0 & 0 & 0 & mn\\ n^{2} & m^{2} & 0 & 0 & 0 & -mn\\ 0 & 0 & 1 & 0 & 0 & 0\\ 0 & 0 & 0 & m & -n & 0\\ 0 & 0 & 0 & n & m & 0\\ -2mn & 2mn & 0 & 0 & 0 & m^{2}-n^{2} \end{array}\right]$$

where

(23) m=cos(α),n=sin(α)

Then, the stiffness contribution from each constituent is assembled while taking into consideration the respective volume fraction of each part within the hybrid 2DTBC. The stiffness of each tow should be determined respectively. The properties of the fibers and matrix used in the stiffness analysis are listed in Table 1.

(24) $$\left[C_{\mathrm{global}}\right]=V_{0}\left[C_{0}\right]+V_{+\alpha }\left[C_{+\alpha }\right]+V_{-\alpha }\left[C_{-\alpha }\right]+V_{\mathrm{matrix}}\left[C_{\mathrm{matrix}}\right]$$

where $V_{0}$, $V_{+\alpha }$, $V_{-\alpha }$, and $V_{\mathrm{matrix}}$ represent the equivalent volume fractions of the four constituents (one axial tow, two braid tows, and one matrix), $V_{0}+V_{-\alpha }+V_{+\alpha }+V_{\mathrm{matrix}}=1$.

### 2.3. Progressive Ply Failure Analysis

**Figure 4:**
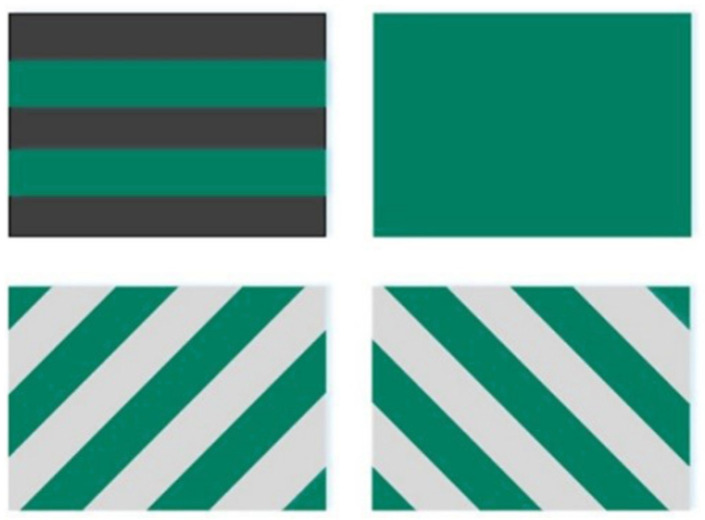
An analytical model consisting of four plies, the ply of carbon fiber tow, two plies of glass fiber tow, and the matrix ply.

As mentioned above, there are four constituents for the hybrid 2DTBC. Each constituent is in turn represented as a ply in the failure analytical model, which is equivalent to a laminate consisted of four plies as shown in Figure 4.

To perform failure analysis of the hybrid 2DTBC, firstly, the global strain {ε} of the structure can be obtained under stress {σ}:

(25) $$\left\{\varepsilon \}={\left[C_{global}\right]}^{-1}\{\sigma \right\}$$

Then, according to the stiffness matrix of each layer, the stress matrix in each ply can be calculated in the global coordinate system (x y z):

(26) $$\left\{\sigma_{0}\right\}=[C_{0}]\{\varepsilon \},\;\;\{\sigma_{+\alpha }\}=[C_{+\alpha }]\{\varepsilon \}\;,\{\sigma_{-\alpha }\}=[C_{-\alpha }]\{\varepsilon \},\{\sigma_{matrix}\}=[C_{matrix}]\{\varepsilon \}$$

Since the strength definition in the failure criterion of the composite materials is defined in the local coordinate system, the stresses of the braid tows in the global coordinate system need to be transformed into the local coordinate system, by rewriting the stress components of braid tows as follows:

(27) $${\left[\sigma \right]}_{xy}=\left\{\begin{array}{ccc} \sigma_{x} & \sigma_{xy} & \sigma_{xz}\\ \sigma_{xy} & \sigma_{y} & \sigma_{yz}\\ \sigma_{xz} & \sigma_{yz} & \sigma_{z} \end{array}\right\}$$

Next, by performing the tensor transformation, the stresses of the braid tows in coordinates ($x^{\prime }$ $y^{\prime }$ $z^{\prime }$) can be expressed by

(28) $${\left[\sigma \right]}_{x^{\prime }y^{\prime }}={\left[R\right]}^{T}{\left[\sigma \right]}_{xy}[R]$$

where

(29) $$[R]=\left[\begin{array}{ccc} \cos(\alpha ) & \sin(\alpha ) & 0\\ -\sin(\alpha ) & \cos(\alpha ) & 0\\ 0 & 0 & 1 \end{array}\right]$$

Performing coordinate transformation again, we can obtain the local stresses in the local coordinate system (1, 2, 3) as shown in Figure 3.

(30) $${\left[\sigma \right]}_{12}={\left[R^{\prime }\right]}^{T}{\left[\sigma \right]}_{x^{\prime }y^{\prime }}\left[R^{\prime }\right]$$

where

(31) $$\left[R^{\prime }\right]=\left[\begin{array}{ccc} \cos(\beta ) & 0 & -\sin(\beta )\\ 0 & 1 & 0\\ \sin(\beta ) & 0 & \cos(\beta ) \end{array}\right]$$

The crimp angle β varies along the wavelength as indicated in Figure 3, which can be treated as a continuous function. Then, the stresses of each ply in the local coordinate system are calculated. By it combining with the Tasi–Wu failure criterion [24] in each ply, the strength analysis can be completed, keeping the external load continuously increasing until Equation (32) is satisfied, so that the 2DTBC fails in the analyzed ply.

(32) $$\begin{array}{l} F_{11}{\sigma_{1}}^{2}+F_{22}{\sigma_{2}}^{2}+F_{33}{\sigma_{3}}^{2}+F_{44}{\sigma_{23}}^{2}+F_{55}{\sigma_{13}}^{2}+F_{66}{\sigma_{12}}^{2}+\\ 2F_{12}\sigma_{1}\sigma_{2}+2F_{13}\sigma_{1}\sigma_{3}+2F_{23}\sigma_{2}\sigma_{3}+F_{1}\sigma_{1}+F_{2}\sigma_{2}+F_{3}\sigma_{3}\geq 1 \end{array}$$

where

(33) $$\begin{array}{l} F_{11}=\frac{1}{X_{T}X_{C}},\;F_{22}=F_{33}=\frac{1}{Y_{T}Y_{C}},\;F_{44}=\frac{1}{{S_{23}}^{2}},\;F_{55}=\frac{1}{{S_{13}}^{2}},\\ F_{66}=\frac{1}{{S_{12}}^{2}},\;F_{12}=F_{13}=-\frac{1}{2}\sqrt{F_{11}F_{22}},\;F_{23}=-\frac{1}{2}\sqrt{F_{22}F_{33}},\\ F_{1}=\frac{1}{X_{T}}-\frac{1}{X_{C}},\;F_{2}=F_{3}=\frac{1}{Y_{T}}-\frac{1}{Y_{C}} \end{array}$$

where *X_T_* and *X_c_* are the longitudinal tensile strength and the longitudinal compressive strength in each ply, respectively, $Y_{T}$ is the transverse tensile strength, $Y_{C}$ is the transverse compressive strength, S is the shear strength, and the subscripts 1, 2, and 3 denote the local coordinates in each ply. The strengths in each ply are determined by the strength of the fiber, the matrix, and their volume fractions [25,26]. The properties of the fibers and matrix in the strength analysis are listed in Table 1.

(34) $$\begin{array}{l} X_{T}=X_{ft}V_{f}+X_{mt}V_{m}\\ X_{C}=X_{fc}\left(V_{f}+E_{m}V_{m}/E_{f_{1}}\right)\\ Y_{T}=\left[1-\left(\sqrt{V_{f}}-V_{f}\right)\left(1-E_{m}/E_{f2}\right)\right]X_{mt}\\ Y_{C}=\left[1-\left(\sqrt{V_{f}}-V_{f}\right)\left(1-E_{m}/E_{f2}\right)\right]X_{mc}\\ S_{12}=S_{13}=\left[1-\left(\sqrt{V_{f}}-V_{f}\right)\left(1-G_{m}/G_{f12}\right)\right]S_{m}\\ S_{23}=S_{f_{23}}V_{f}+S_{m}V_{m} \end{array}$$

where *T* and *C* denote tension and compression, the subscripts *f* and *m* indicate the fiber and matrix, respectively, and V is the volume fraction. Then, all the coefficients in the Tsai–Wu criterion can be obtained based on the mechanical properties and volume fraction of the fiber and matrix. The failure modes of each ply can be divided into two types: fiber failure and matrix failure [11]. We assume that even if the matrix is damaged, such as cracks in the matrix, the fiber can still withstand the mechanical loads in the fiber direction, because if the entire ply is considered to fail, the strength of the 2DTBC will be underestimated. Here, we conducted a standard progressive ply failure analysis and performed the following steps sequentially:

(1) Increase the external load until one of the four plies fails according to the Tsai–Wu criterion;
(2) Recalculate the local stiffness of this ply and the global stiffness matrix using degraded elastic constants determined using different failure modes;
(3) Determine the local stress of each ply again from Equation (30);
(4) Continue to increase the load gradually until a new ply fails or the failure mode of the failed ply changes from the fiber failure to matrix failure;
(5) Repeat steps 2–4 until all four plies of the 2DTBC fail;
(6) The maximum load is extracted as the ultimate strength of the 2DTBC.

Finally, we compared the predicted results of the analytical model with experimental data. The braid composite material was piled up with two 2DTBCs along the same direction, and the average thickness of each layer was measured to be 2 mm. Other experimental details are given in [15], and the properties of the carbon fiber, glass fiber, and matrix are listed in Table 1. The comparison results are listed in Table 2, which shows that the accuracy of the analytical model is acceptable.

## 3. Optimization

### 3.1. Model Description

**Figure 5:**
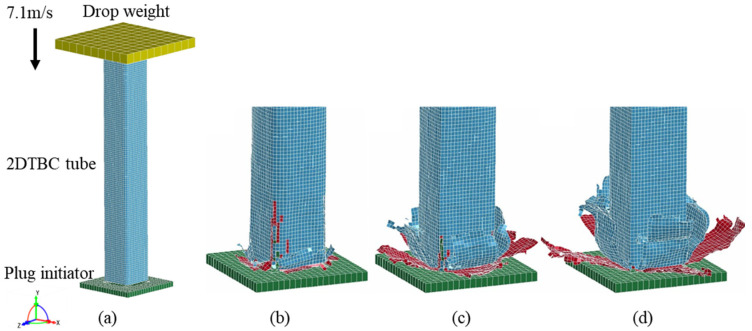
(**a**) FE model for composite tube crush; the crushing state of the 2DTBC tube when displacement is 10 mm (**b**), 30 mm (**c**), and 70 mm (**d**).

The analytical model of the 2DTBC was applied to evaluate the stiffness and strength of the 2DTBC with different material structure parameters. To evaluate the crashworthiness capacity and reduce the mass and cost of a 2DTBC tube under axial impact, the finite element model of the thin-wall-tube similar to that of ref. [27] was established, and collapse simulations were carried out in the commercial software LS-DYNA. As shown in Figure 5a. The structure considered in this study consists of three portions: a two-dimensional tri-axial braided tube, a drop weight, and a plug-type initiator.

The drop weight and initiator were modeled as rigid with an equivalent weight. The orientation of the carbon tow is the same as the crash direction of the tube. To allow for delamination failure, the two-dimensional tri-axial braided tube was modeled with two layers of shell elements connected through the Tiebreak contact definition of LS-DYNA [28]. The default element formulation, Belytschko–Tsay, was employed for the analysis. For the boundary conditions, the drop weight was joined to the tube through constrained nodes. The rigid initiator was fixed, and the drop weight was released at an initial velocity of 7.1 m/s. For the simulation of specimens, we employed the efficient progressive failure model MAT58 in LS-DYNA, which utilizes the Hashin–Rotem failure criterion [29] to assess the failure of each ply. This failure criterion has been validated to effectively simulate impact failure in woven composite materials [27].

Figure 5b–d shows the comparison of the crush state of the 2DTBC tube at displacements of 10 mm, 30 mm, and 70 mm, respectively. It can be seen that during impacting, the braided composites exhibited fiber curling and delamination, consistent with the experimental results reported in Ref. [27]. Composite tube failures under compression can be categorized into stable and unstable failures. Unstable failures include buckling, penetration, and roll abrasion. Stable failures are further divided into fiber delamination and debonding along the axis, fiber crushing, and brittle fiber destruction. Stable failure modes primarily absorb energy by converting collision forces into the fiber matrix fracture, friction, composite layer bending, and crack propagation, thereby improving the specific energy absorption (SEA) of the tube. In contrast, unstable failures lead to excessively high peak loads, which negatively impact crash safety [30,31]. Therefore, increasing the proportion of stable failure modes is essential for improving the crash performance of composite tubes during compression collapse.

### 3.2. Definition of the Crashworthiness Optimization Problem

#### 3.2.1. Design Variables and Objective Functions

The structural impact problem is one of the problems with geometric and material nonlinearities. It is expected that the braided tube can absorb as much energy as possible in a unit specimen mass. The energy absorption per unit structural weight is defined as

(35) $$SEA=\frac{E_{\mathrm{total}}}{m}$$

where *m* is the total mass of the tube considered, and $E_{\mathrm{total}}$ is referred to the area under the force–displacement curve:

(36) $$E_{\mathrm{total}}=\int_{0}^{\delta }G(z)dz$$

where G(z) represents the strain energy density of the structure. The peak crushing force *P* is considered to be one of the important design objectives to prevent the occupant’s body from severe biomechanical injury [32,33]. The peak crushing force *P* is defined as

(37) P=max{F(z)∀z∈[0,δ]}

where δ is the total axial crushing distance, and F(z) is the value of the crushing force at the crushing length z.

In addition, lightweight design plays a crucial role in industrial applications. The efficient density of the hybrid 2DTBC can be evaluated based on the homogenization process.

(38) $$\rho =\rho_{m}\left(1-V_{ft}\right)+\rho_{c}V_{ft}V_{cr}+\rho_{g}\left(V_{ft}-V_{ft}V_{cr}\right)$$

where $V_{ft}$ is the total fiber volume fraction, $V_{cr}$ is the relative volume fraction of the carbon fiber in the total fiber volume fraction, and $\rho_{c}$, $\rho_{g}$, and $\rho_{m}$ are the density of the carbon fiber, glass fiber, and matrix, respectively. The density of the components is presented in Table 3.

Furthermore, the material cost is an essential factor to be considered in most applications of composites. Compared with the carbon fiber, the glass fiber has higher toughness and a lower cost. In this work, the carbon fiber was used as the axial tow, and the glass fiber was selected as braid tows. The hybrid effect of different fibers may be helpful to improve the crashworthiness and structural integrity of the 2DTBC. For the hybrid 2DTBC, the cost function can be expressed as follows:

(39) $$c=c_{m}\rho_{m}\left(1-V_{f}\right)+c_{c}\rho_{c}V_{f}V_{cr}+c_{g}\rho_{g}\left(V_{f}-V_{f}V_{cr}\right)$$

where $c_{c}$, $c_{g}$, and $c_{m}$ are the cost per volume of the carbon fiber, glass fiber, and matrix, respectively. The cost for each component is shown in Table 3.

The total fiber volume fraction *V_ft_* and the relative volume fraction of the carbon fiber in the total fiber volume fraction *V_cr_* have a high impact on the weight and cost of the final design according to Equations (38) and (39), while the braid angle plays an important role to control the mechanical performances of the hybrid 2DTBC. Thus, the design variables chosen for this study are as follows:

- *V_ft_*: The total fiber volume fraction.
- *V_cr_*: The relative volume fraction of carbon fiber in the total fiber volume fraction.
- α: The braid angle.

In addition, to determine the mechanical performances of the hybrid 2DTBC based on CCM and Quek’s model, it is necessary to know the volume fractions of the four parts, $V_{0}$, $V_{+\alpha }$, $V_{-\alpha }$, and *V_matrix_* (volume of axial tow, two braid tows, and matrix). The inherent relationship between design variables and the volume fractions of the four parts can be expressed as follows:

(40) $$V_{0}=\frac{V_{ft}V_{cr}}{V_{f}^{0}}$$

(41) $$V_{+\alpha }=V_{-\alpha }=\frac{V_{ft}-V_{ft}V_{cr}}{2V_{f}^{1}}$$

where $V_{f}^{0}$ and $V_{f}^{1}$ are the local fiber volume fraction in the axial tow and the braid tows, respectively. It should be noted that $V_{f}^{0}$ and $V_{f}^{1}$ have no relation to the design variables and can only be measured through experiments or using geometrical models. Thus, they are treated as uncertain but bounded variables. According to the material supplier’s parameters, the values of $V_{f}^{0}$ and $V_{f}^{1}$ varied in the range of 0.6–0.7 and 0.8–0.9, respectively. Table 4 gives insight to the upper and lower bounds of each design variable.

#### 3.2.2. Problem Formulations

In industrial applications, achieving optimal results often requires satisfying multiple objectives simultaneously. Crashworthiness optimization plays an important role in the performance improvement design of the composite structures. To account for these two different design criteria, in this paper, the optimization of crashworthiness and lightweight design were separately explored. In the crashworthiness optimization, we examine whether improvements in crashworthiness lead to significant increases in density and cost. Additionally, in the lightweight cost optimization, crashworthiness is treated as a constraint, ensuring that material density and cost are reduced while maintaining the required crash performance. This approach allows us to address both design criteria effectively and achieve an optimal balance in practical applications. The optimization problem can be mathematically formulated as follows:

(1) Crashworthiness optimization issues:

(42) $$\{\begin{array}{l} \min\left\{-SEA\left({\tilde{V}}_{ft},{\tilde{V}}_{cr},\tilde{\alpha }\right),P\left({\tilde{V}}_{ft},{\tilde{V}}_{cr},\tilde{\alpha }\right)\right\}\\ s.t.\;0.3<{\tilde{V}}_{ft}<0.7\\ \;\;\;\;\;\;\;0.1<{\tilde{V}}_{cr}<0.9\\ \;\;\;\;\;\;\;10<\tilde{\alpha }<85 \end{array}$$

**Figure 6:**
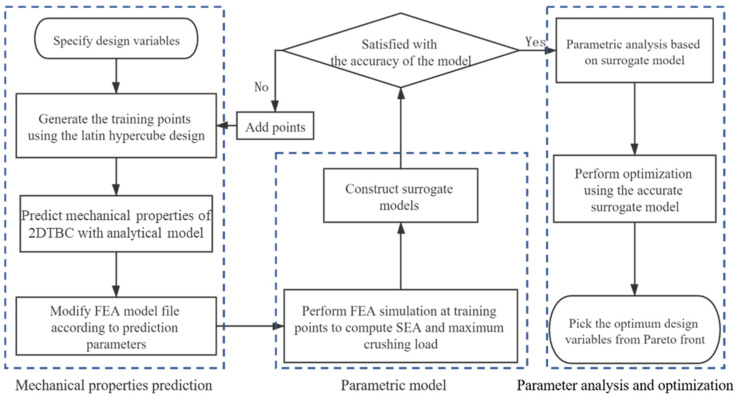
A flow chart of the integrated crashworthiness optimization design process.

(2) Lightweight design of structure:

(43) $$\{\begin{array}{l} \;\min\left\{\rho \left({\tilde{V}}_{ft},{\tilde{V}}_{cr},\tilde{\alpha }\right),c\left({\tilde{V}}_{ft},{\tilde{V}}_{cr},\tilde{\alpha }\right)\right\}\\ s.t.P\left(M_{S}\left({\tilde{V}}_{ft},{\tilde{V}}_{cr},\tilde{\alpha }\right)\geq M_{S0}\right)\geq 95\%\\ \;P\left(M_{P}\left({\tilde{V}}_{ft},{\tilde{V}}_{cr},\tilde{\alpha }\right)\geq M_{P0}\right)\geq 95\% \end{array}$$

where ρ and c represent the efficient density and the price per cubic centimeter of the hybrid 2DTBC. $M_{S}$ and $M_{P}$ are the means of the energy absorption per unit structural weight and the peak crushing force, respectively. $M_{S0}$ and $M_{P0}$ are the constraints in the engineering application. Meanwhile, reliability needs to be evaluated as an implicit constraint to ensure structure security. Thus, the material–structure integration multi-objective optimization is proposed and employed to determine the optimal combination of the design parameters. Figure 6 illustrates the flow chart of the multi-objective optimization design method. Firstly, sample points for braiding parameters are generated in the design space using Latin hypercube sampling, and the corresponding elastic and strength performance parameters are computed using the performance prediction analytical model developed in Section 2. The finite element model file is then updated, and the mechanical response related to the crash performance at each sample point is calculated using the LS-DYNA solver. A Kriging surrogate model is constructed based on the sample set, and its accuracy is validated. If the accuracy does not meet the required specifications, additional iterations are performed. Ultimately, a predictive model is developed to correlate fine-scale parameters with the macroscopic crash mechanical properties of 2DTBC. A multi-objective optimization problem is then formulated and solved.

### 3.3. Solution

In the field of crashworthiness optimization, population-based heuristic multi-objective evolutionary algorithms, such as MOEA/D, SPEA-II, and NSGA-II, have become the most widely used methods due to the unknown gradient information when using FEM simulations. In this paper, NSGA-II [34] was used as a multi-objective optimization solver to cope with the problem described above due to its effectiveness and easy implementation. In addition, the Pareto-sets can be obtained, which can provide a series of optimal solutions for further analysis.

However, the direct use of non-linear FEA to evaluate the objective and the constraint values would be unaffordable and inefficient. In the numerical approaches for multi-objective optimization, surrogate models based on a global approximation strategy are more computationally efficient and gradient-free. To reduce the calculation cost, the Kriging model was chosen as the surrogate model [35]. The global approximation model in the Kriging model can be expressed as follows:

(44) $$y(x)=\sum_{j=1}^{p}\beta_{j}f_{j}(x)+z(x)=f{\left(x\right)}^{T}\beta +z(x)$$

where β stands for the corresponding undetermined parameter, f(x) is the column vector of polynomial functions, z(***x***) is a stochastic process with mean zero, and the variance and covariance matrix of z(***x***) is expressed as

(45) $$\mathrm{cov}\left[z\left(x_{i}\right),z\left(x_{j}\right)\right]=\sigma^{2}R\left[R\left(x_{i},x_{j}\right)\right]$$

where *R* is the correlation matrix of sample points. $R\left(x_{i},x_{j}\right)$ represents the correlation function between *x_i_* and *x_j_*. The Gaussian correlation function is usually used as the correlation function and can be expressed as follows:

(46) $$R\left(x_{i},x_{j}\right)=\exp\left[-\sum_{k=1}^{m}\theta_{k}{\left|x_{i}^{k}-x_{j}^{k}\right|}^{2}\right]$$

where *m* is the dimension number of the design variables, $-\sum_{k=1}^{m}\theta_{k}{\left|x_{i}^{k}-x_{j}^{k}\right|}^{2}$ is the kernel function, and θ is the correlation length, which is used to fit the surrogate model. The predictor value $\hat{y}(x)$ is predicated at any unknown points *x* as

(47) $$\hat{y}(x)=f{\left(x\right)}^{T}\hat{\beta }+r{\left(x\right)}^{T}R^{-1}(Y-F\hat{\beta })$$

where

(48) $$F={\left[f{\left(x_{1}\right)}^{T},f{\left(x_{2}\right)}^{T},\cdots ,f{\left(x_{n}\right)}^{T}\right]}^{T}$$

(49) $$r(x)={\left[R\left(x,x_{1}\right),R\left(x,x_{2}\right),\cdots ,R\left(x,x_{n}\right)\right]}^{T}$$

(50) $$\hat{\beta }={\left(F^{T}R^{-1}F\right)}^{-1}F^{T}R^{-1}Y$$

where $Y={\left[y_{1},y_{2},\cdots ,y_{n}\right]}^{T}$ is the output value obtained via simulation and *n* is the training sample size. F is a unit matrix, and $\hat{\beta }$ is estimated using least squares regression as shown in Equation (50).

## 4. Results and Discussion

### 4.1. Error Evaluation of the Surrogate Model

**Figure 7:**
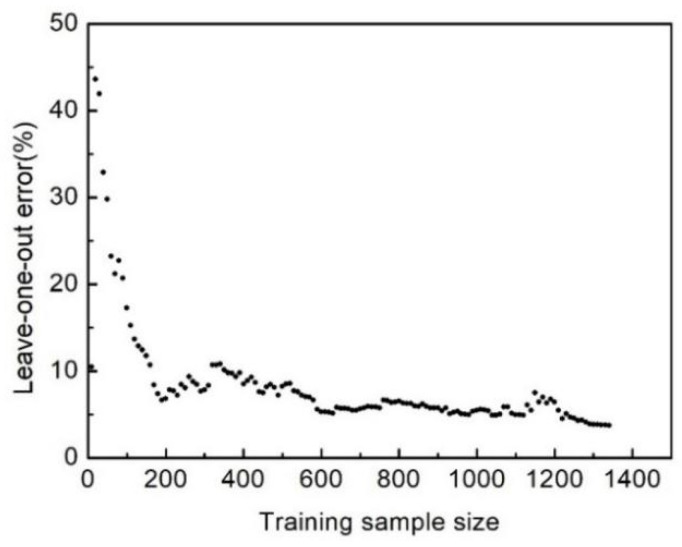
Leave-one-out error versus training sample size for SEA.

**Figure 8:**
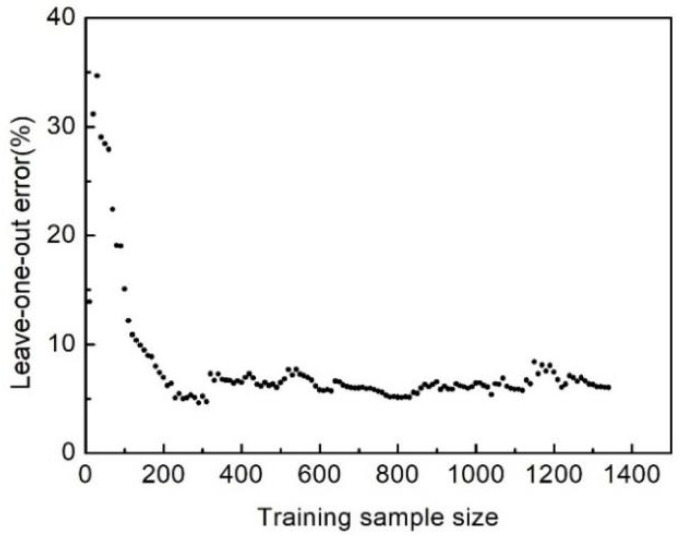
Leave-one-out error versus training sample size for peak load.

To measure the accuracy of the Kriging models, a leave-one-out error estimation [36] was adopted. The evaluation function is expressed as follows:

(51) $$e_{LOO}=\sqrt{\frac{1}{N}\sum_{i=1}^{N}\frac{{\left(y_{i}-{\hat{f}}^{-i}\left(x_{i}\right)\right)}^{2}}{y_{i}^{2}}}$$

where *N* is the number of the samples, $y_{i}$ is the exact system responses at the sampling points, and ${\hat{f}}^{-i}\left(x_{i}\right)$ is the predictor value at sample point *x_i_*. In this study, the crashworthiness capacity is evaluated with the value of SEA and *P*, which are evaluated using the two independent Kriging models. Figure 7 and Figure 8 show the leave-one-out errors of the Kriging models with an increase in samples. When the number of sampling points reaches 1350, the leave-one-out errors of SEA and *P* are 3.77% and 6.04%, respectively, suggesting that the accuracy of the model can be acceptable.

### 4.2. Parametric Analysis

**Figure 9:**
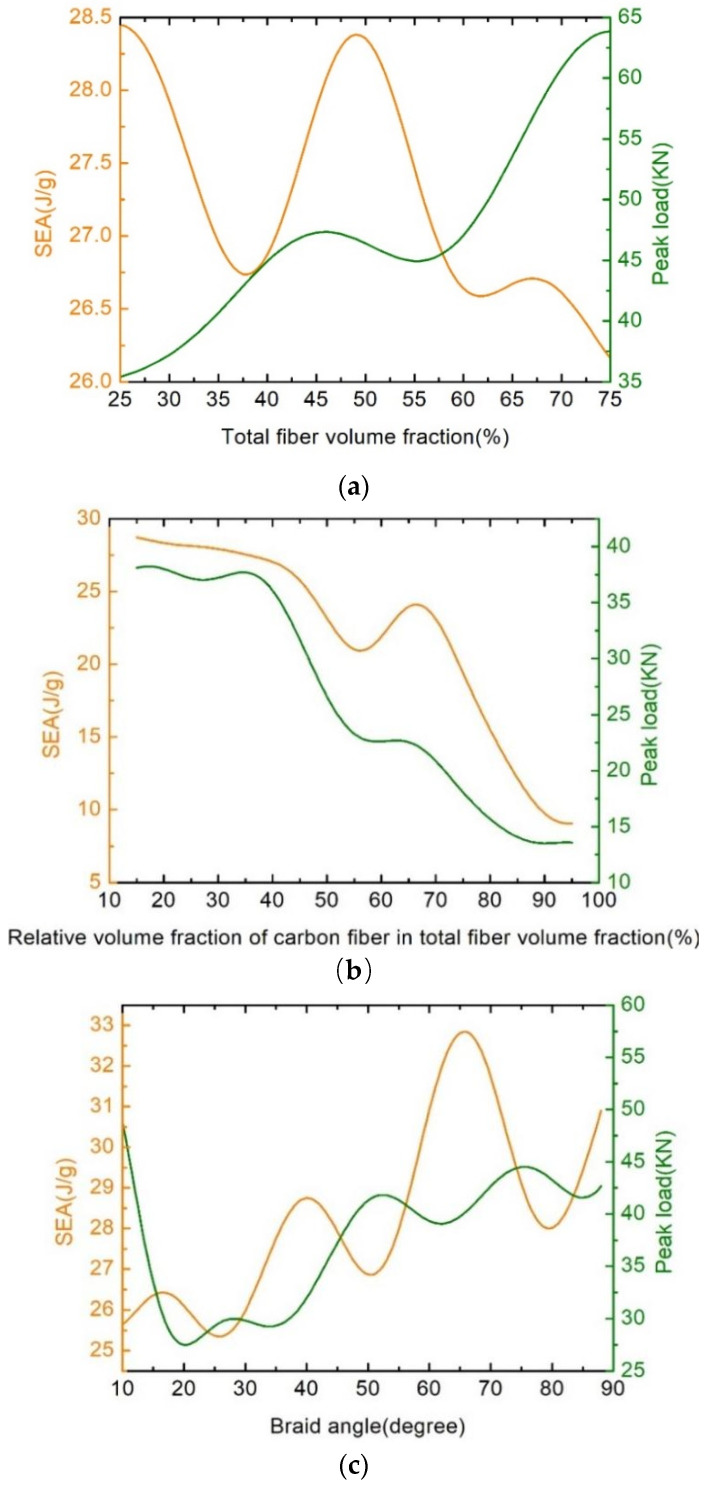
Variations in the crashworthiness capacity with total fiber volume fraction (**a**), relative volume fraction of carbon fiber in total fiber volume fraction (**b**), braid angle (**c**).

Building a surrogate model that can reflect the highly non-linear mapping between the influence factors and crashworthiness response is necessary for this study. To analyze the influence of the parameters, a variable-controlling approach has been applied. The relationships concerning SEA and *P* are plotted as functions of *V_ft_*, *V_cr_*, and α in Figure 9.

It can be seen from Figure 9a that the value of *P* increases gradually with the increase in *V_ft_*, while SEA exhibits an approximate sinusoidal fluctuation, varying between 26.25 J/g and 28.5 J/g. Since the axial impact was mainly applied to the fiber tows, a higher fiber volume will lead to higher axial strength. On the other hand, it can be seen from Equations (35)–(38) that the relationship between *V_ft_* and SEA is nonlinear, so SEA shows regular fluctuation. In Figure 9b, it can be seen that the crashworthiness performance of the hybrid 2DTBC is decreased with the increase in *V_cr_*. It can be explained from the microstructure of the hybrid 2DTBC. As shown in Figure 1, the carbon fiber tows mainly bear the load of the axial collision. Since the total fiber volume fraction is fixed, the volume fraction of glass fiber tows decreases with the increase in *V*_cr_. Because glass fiber tows mainly bear the load in the transverse direction, the lack of glass fiber tows in the transverse direction will cause unstable damage, which is characterized by an initial load peak followed by a catastrophic failure, like buckling, interpenetration, or barreling [37,38,39,40], eventually leading to a reduction in SEA. The crashworthiness performances follow a fluctuating ascending trend as shown in Figure 9c. Since the transverse strength of the 2DTBC increases with the increase in the braid angle, the dominant failure mode of this structure will be a stable failure mode, like fiber splaying, fragmentation, and brittle fracture.

### 4.3. Pareto Optimal Solutions

**Figure 10:**
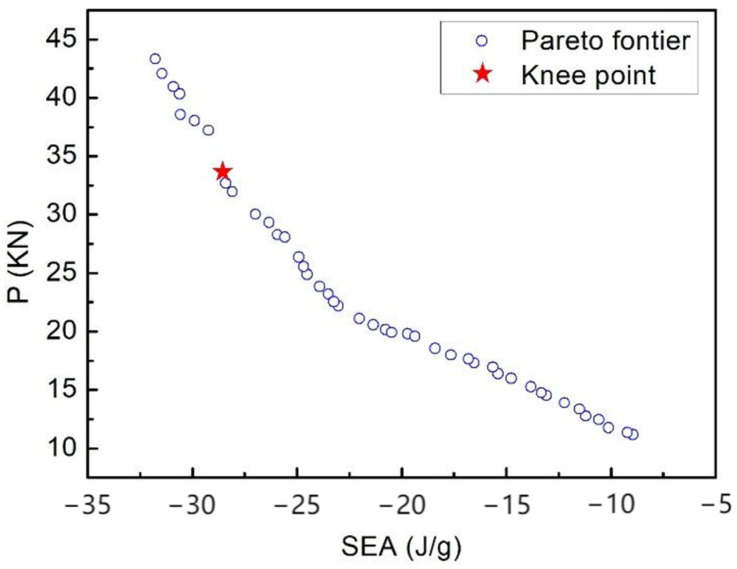
Pareto front for the absorbed energy and the peak load.

For crashworthiness optimization, the Pareto set, as shown in Figure 10, was obtained by employing NSGA-II as the optimization solver. It can be seen that there is competition between SEA and *P*. In other words, for any improvement in one objective, the other objective must be compromised. Although the Pareto front with a range of optimal solutions is crucial for the designer, further decisions must be made for the most satisfactory solution (Knee Point) from the Pareto front according to the designer’s preference for the objective functions. In this paper, the Knee Point was determined through normalization, which can improve the accuracy of the distance from Knee Point to a “Utopia Point” [34]. The determined Knee Point is shown in Figure 10.

Table 5 shows the comparison results of design variables between the optimal model and baseline model and the simulated values of the crashworthiness capacity corresponding to the optimal combination of design variables. The initial design has been optimized, which has a *p*-value that is 16.45% smaller and an SEA value that is 15.93% higher compared with those of the original design. The material cost and density are 112.34 $/cm^3^ and 1.48 g/cm^3^, respectively. The crashworthiness capacity of the 2DTBC has been greatly improved after optimization, while the corresponding material cost and density have not increased sharply with the improvement in impact performance. Based on the analysis in Section 4.2, it can be seen that the algorithm tends to reduce the use of *V_ft_* and increase the amount of glass fiber tows, thereby decreasing P and enhancing the transverse load-bearing capacity of the 2DTBC, which helps stable failure modes.

**Figure 11:**
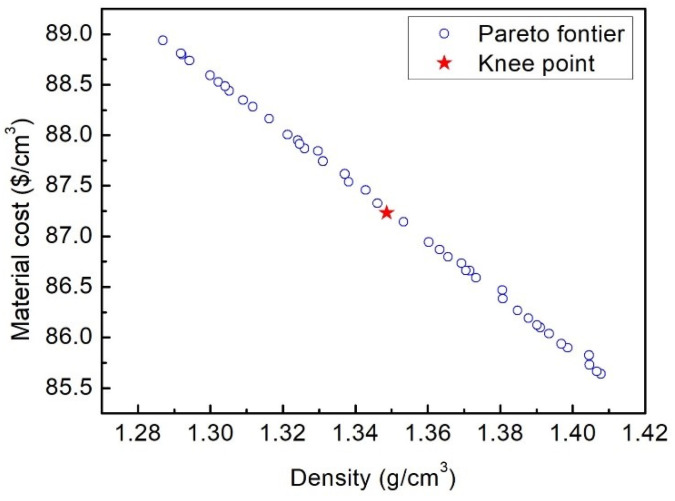
Pareto front for material cost and density.

For the lightweight optimization process of the 2DTBC, density and cost minimization are the objectives while meeting the crashworthiness requirement. Figure 11 illustrates the Pareto sets of the optimization. The result of the lightweight design is compared to the initial design, as shown in Table 6. On the premise of meeting the crashworthiness capacity, the cost and density of the 2DTBC decreased by 28.37% and 6.72%, respectively. It should be pointed out that to reduce the cost of the 2DTBC, the total fiber volume fraction and the relative volume fraction of the carbon fiber in the total fiber volume fraction also decreased compared with those during the crashworthiness optimization. On the other hand, the braid angle remained at about $45^{\circ }$ in both typical optimization problems. This suggests that $45^{\circ }$ is the most economical braid angle of the 2DTBC under axial crash.

## 5. Conclusions

In this study, we developed a framework for evaluating and the optimal design of the macroscopic crashworthiness of 2D braided composites (2DTBCs) by integrating mesostructure analysis with material, structural performance, and multi-objective optimization. The research demonstrated that significant enhancements in 2DTBC’s crashworthiness can be achieved through optimization. Specifically, we employed a prediction algorithm based on the classical composite mechanics (CCM) and an analytical laminate model within finite element analysis, supported by Kriging surrogate models to enhance computational efficiency. Our findings indicate that a strategic reduction in the fiber volume fraction (V*_ft_*) combined with an increased proportion of glass fiber tows in the hybrid 2DTBC not only improves its crashworthiness but also contributes to weight and cost reductions. This approach facilitates the stabilization of failure modes, leading to more predictable and reliable performance under impact conditions. Moreover, the optimization revealed that a braid angle of 45° is the most effective for enhancing the impact resistance of the hybrid 2DTBC, balancing structural integrity and material efficiency. Overall, the results underscore the effectiveness of the proposed optimization framework in enhancing the crashworthiness and economic efficiency of the 2DTBC, paving the way for more cost-effective and high-performance composite materials in impact applications.

## Figures and Tables

**Table 1:** Properties of the carbon fiber, glass fiber, and matrix.

Parameter	Value	Parameter	Value
Carbon longitudinal Young’s modulus	255 Gpa	Matrix (epoxy) Young’s modulus	3.056 Gpa
Carbon transverse Young’s modulus	15 Gpa	Matrix Poisson’s ratio	0.3
Carbon longitudinal shear modulus	24 Gpa	Matrix tensile strength	74 MPa
Carbon transverse shear modulus	5 Gpa	Matrix compression strength	241 Mpa
Carbon longitudinal Poisson’s ratio	0.14	Matrix shear strength	60 Mpa
Carbon longitudinal tensile strength	4400 Mpa	Carbon fiber volume fraction	39.2%
Carbon longitudinal compression strength	2470 Mpa	Glass fiber volume fraction	12.8%
Glass Young’s modulus	73 Gpa	Matrix volume fraction	48%
Glass Poisson’s ratio	0.18	Braid angle	$45^{\circ }$
Glass tensile/compression strength	2600 Mpa	Braid tow amplitude	0.701 mm
Glass shear strength	50 Mpa	Braid tow wavelength	14.03 mm

**Table 2:** Comparison of prediction results of the analytical model and experimental results.

	*E*_xx_/GPa	*E*_yy_/GPa	*G_xy_*/GPa	*ν*_12_	*X_t_*/MPa	*X_c_*/MPa	*Y_t_*/MPa	*Y_c_*/MPa	*S_xy_*/MPa
Analytical model	76.4	8.29	5.37	0.32	1280	392.4	52.3	122	97
Experiments	74.5	9.65	5.74	0.29	1090	416.2	40.8	125.4	91.2
Relative error	2.5%	14.1%	6.4%	10.3%	17.4%	5.7%	14.8%	2.7%	6.3%

**Table 3:** The density and cost of carbon fiber, glass fiber, and matrix.

	Carbon Fiber	Glass Fiber	Matrix
Density (kg/m^3^)	1800	2460	1090
Cost ($/Kg)	35.9	24.5	6.2

**Table 4:** The bound value of design variables.

Design Variables	Lower Bound	Upper Bound
*V_ft_*	0.3	0.7
*V_cr_*	0.1	0.9
α	10	85

**Table 5:** Results before and after crashworthiness optimization.

	*V_ft_*	*V_cr_*	α	SEA (J/g)	P (KN)
Initial design	0.52	0.75	45	23.23	36.64
Crashworthiness Optimization	0.43	0.32	43.89	28.52	30.58
Improvement				22.9%	16.45%

**Table 6:** Results before and after lightweight design.

	*V_ft_*	*V_cr_*	α	Material Cost ($/cm^3^)	DenSity (g/cm^3^)
Initial design	0.52	0.75	45	121.80	1.43
Lightweight design	0.33	0.51	44.62	87.25	1.35
Improvement				28.37%	6.72%

## Data Availability

The original contributions presented in the study are included in the article, further inquiries can be directed to the corresponding author.
